# Ursolic Acid Reverses the Chemoresistance of Breast Cancer Cells to Paclitaxel by Targeting MiRNA-149-5p/MyD88

**DOI:** 10.3389/fonc.2019.00501

**Published:** 2019-06-14

**Authors:** Fenfen Xiang, Yan Fan, Zhenhua Ni, Qiaoli Liu, Zhaowei Zhu, Zixi Chen, Wenbin Hao, Honghong Yue, Rong Wu, Xiangdong Kang

**Affiliations:** ^1^Laboratory Medicine, Putuo Hospital, Shanghai University of Traditional Chinese Medicine, Shanghai, China; ^2^Central Laboratory, Putuo Hospital, Shanghai University of Traditional Chinese Medicine, Shanghai, China

**Keywords:** ursolic acid, paclitaxel, breast cancer, resistance, miR-149-5p, MyD88

## Abstract

Paclitaxel (PTX) is widely used as a front-line chemotherapy for breast cancer treatment. However, its clinical applications are limited by the development of chemoresistance. The objective of this study was to investigate the reversal effects of ursolic acid (UA) on PTX resistance and the possible mechanisms in breast cancer. The role of miRNA-149-5p/MyD88 in the regulation of PTX resistance was investigated by the transfection of breast cancer cells with MDA-MB-231 (231) and MDA-MB-231/PTX-resistance (231/PTX) with lentiviruses carrying the MyD88 gene, shRNA specific for MyD88, the miR-149-5p gene, and shRNA specific for miR-149-5p. The PTX sensitivity was assessed by a CCK-8 assay. qRT-PCR and Western blot analyses were used to detect changes in the mRNA and protein levels. Flow cytometry was used to measure the rate of cell apoptosis. A luciferase activity assay was used to detect the binding site of miR-149-5p on the 3′UTR of MyD88. 231/PTX cells were injected into the flanks of female athymic nude mice, and the mice were randomly divided into the five following groups: PBS, PTX (low), PTX (high), UA, and PTX+UA. Our data show that UA reversed the resistance of breast cancer 231/PTX cells to PTX *in vitro* and *in vivo*. UA treatment significantly increased the expression of miR-149-5p, which was lower in 231/PTX cells than in 231 cells. Furthermore, the overexpression of miR-149-5p increased the sensitivity of 231/PTX cells to PTX treatment, whereas the knockdown of the miR-149-5p gene attenuated the effects of UA on the regulation of PTX sensitivity. A luciferase assay demonstrated that miR-149-5p could directly regulate the transcriptional activity of MyD88, a known PTX-resistance gene, by targeting the 3′UTR of MyD88. Meanwhile, the downregulation of MyD88 through the overexpression of miR-149-5p or UA treatment inhibited the activation of the Akt signaling pathway in 231/PTX cells. Thus, our data indicate that UA can reverse PTX resistance by targeting the miRNA-149-5p/MyD88 axis in breast cancer cells.

## Introduction

Breast cancer is one of the most common clinical cancers in the world, and it is also the main cause of cancer-related death in women (1). As a microtubule stabilizer with good tolerability, PTX is widely used to treat breast cancer; however, its therapeutic efficacy is limited, due to the development of resistance (2). Thus, a better understanding of the mechanisms underlying PTX resistance and attempts to reverse their resistance effectively are crucial for improving patients' treatment options and prognosis.

Our previous work (3) demonstrated that the expression of MyD88 was increased in breast cancer tissues, and its expression level was correlated with PTX resistance. Furthermore, our data showed that the downregulation of MyD88 expression could significantly enhance PTX chemosensitivity in both breast cancer MCF-7 cells, lung cancer A549 cells (4), and ovarian cancer A2780 cells (5). Therefore, searching for molecules or drugs that can effectively inhibit the expression of MyD88 can help reverse PTX resistance.

MicroRNAs (miRNAs) are single-stranded non-coding RNAs that silence genes by binding to the complementary sequences located in the 3′UTRs of their target mRNAs (6, 7). MiRNAs are hypothesized to regulate up to 30% of human gene expression, although they account for only ~1% of all human genes (8–12). Our previous study showed that the downregulation of miR-149-5p increased the level of MyD88, and consequently, PTX chemosensitivity was significantly decreased (6). It was also reported that miR-149 can directly target the 3′UTR of MyD88 and the posttranscriptional regulation of MyD88 protein expression, which indicated that miR-149 may be a key regulator in macrophage TLR/MyD88 signaling (13). However, it remains unclear whether miR-149 can regulate PTX resistance in breast cancer by regulating MyD88 transcriptional activity.

UA is a pentacyclic triterpene acid that is widely distributed in medicinal and edible plants, such as apples and other waxy shell fruits (14). Recent studies (15–19) and our previous research (20) have demonstrated that UA can inhibit the growth of many human cancer cell lines, such as those of breast cancer, gastric cancer, liver cancer, and skin cancer. UA is considered a potential chemotherapeutic agent suitable for cancer treatment. Additionally, research has shown that UA attenuates PTX resistance through multiple pathways (21). Our preliminary study showed that MyD88 was implicated in PTX sensitivity in breast cancer and that UA increased the expression of miR-149-5p and subsequently decreased the expression of MyD88.

Therefore, the aim of this study was to further elucidate the reversal effects of UA on PTX chemoresistance and the possible mechanisms in breast cancer. Human breast cancer cell lines, 231, and 231/PTX, were utilized to investigate the antitumor effects of UA with regards to PTX treatment *in vitro* in human breast cancer cells. Breast cancer xenografts of nude mice were selected for *in vivo* studies. Our work indicates that UA could reverse PTX resistance in breast cancer by modulating miR-149-5p and MyD88 expression, which sheds light on the improvement of breast cancer chemotherapy and provides evidence for further clinical investigation.

## Materials and Methods

### Cell Cultures

Human MDA-MB-231 and MDA-MB-231 PTX-resistant cell lines (obtained from Shanghai Gene Biochemistry Co., Ltd.) were maintained in Leibovitz's L-15 Medium (Gibco Industries, Inc.) with 10% fetal bovine serum at 37°C in a humidified atmosphere.

### Cell Proliferation Assays

The cell proliferation was measured by using a Cell Counting Kit-8 (CCK-8, Dojindo, Japan) to generate a growth curve. The cells were seeded at 0.6 × 10^4^ cells per well in a 96-well plate and were incubated overnight. The cells were then treated with various concentrations (0, 5, 10, 20, 40, 80, 160, and 320 μM) of PTX (MedCham Express, dissolved in DMSO), with or without UA (20 μM, Selleck, Houston, United States) for 48 h, and the appropriate controls were treated with DMSO at the same concentrations. The cell proliferation per well was determined by CCK-8 solution, and the optical density was measured at 450 nm.

### RNA Extraction and Quantitative Real-Time PCR (qRT-PCR)

The total mRNA was isolated using the TRIzol Reagent Kit, and the PrimeScript RT Reagent Kit (Takara Bio, Inc.) was used for reverse transcription. The miRNA was extracted using the miRNA Extraction Kit (Tiangen Bio, Shanghai, China), and the expression of mature miRNAs was assayed using stem-loop RT. The gene expression level was measured by a qRT-PCR system (StepOne Plus; Applied Biosystems, USA). GAPDH and U6 snRNA were used to normalize the relative amount of each target gene or each miRNA separately. The relative expression was calculated by the 2^−ΔΔCt^ method. The primers used are shown in Table 1.

**Table 1:** Nucleotide sequences of primers used for qRT-PCR reactions.

**Gene**	**Forward**	**Reverse**
GAPDH	5′-ATGCTGCCCTTACCCCGG-3′	5′-TTACTCCTTGGAGGCCATGTAGG-3′
MYD88	5′-AAAGGCTTCTCAGCCTCCTC-3′	5′-ACTGCTCGAGCTGCTTACCA-3′
BAX	5′-CAGATCATGAAGACAGGGGCC-3′	5′-GCCCACGTCCCCCAATCC-3′
BCL-2	5′-CTTACTAATAACGTGCCTCATGAAATAAAGATCCG-3′	5′-TCCCAGCCTCCGTTATCCTGGA-3′
MiR-149-5p	5′-TCTGGCTCCGTGTCTTCACTCCCA-3′	
U6	CD201-1045(obtained from Tiangen Biotech)	

### Western Blot Analysis

We lysed the cells using a protein extraction reagent (Beyotime, Jiangsu, China) in the presence of protease inhibitor, and the protein concentration was measured using a BCA Protein Assay Kit (Beyotime, Jiangsu, China). Soluble lysates containing ~50 μg proteins per sample were resolved by SDS/PAGE gel and transferred to a PVDF membrane (Merck Millipore). Blocking was performed for 2 h with 5% fat-free milk in TBST, and the membranes were incubated with primary antibodies against β-actin (Beyotime), MyD88 (CST), Akt (CST), PAkt (CST), PI3K (CST), Bax (CST), and Bcl-2 (CST) overnight at 4°C; then, the membranes were incubated with secondary antibodies (1:1000) at room temperature for 1 h. After extensive washing with TBST, the immunoblot was detected with enhanced chemiluminescence (Pierce Biotechnology).

### Apoptosis Assay

After drug treatment for 48 h, the 231 and 231/PTX cells were collected and suspended in binding buffer and then stained with Annexin V-Phycoerythrin (BD Biosciences) for 15 min at room temperature in the dark. Subsequently, the cells were analyzed by flow cytometry using Calibur (BD Biosciences) within 1 h.

### Construction of the MyD88 and miR-149-5p Lentiviral

The human MyD88 cDNA and siRNA sequences against MyD88 were synthesized by GenePharma (Shanghai, China), and the approach was described as previously reported (4). The overexpression constructs of MyD88 and the control were designated MyD88-OE and MyD88-NC, and the knockdown of MyD88 and the control were designated MyD88-KD and MyD88-NC, respectively. The siRNA sequences against miRNA-149-5p (5′-GGGAGUGAAGACACGGAGCCAGA-3′) were constructed with the LV3-pGLV-GFP/puro lentiviral by GenePharma (Shanghai, China), and the whole gene of miRNA-149-5p synthesized by Gene (Shanghai, China) was subcloned into the hU6-MCS-Ubiquitin-EGFP/Puro lentiviral vector.

### Dual-Luciferase Reporter Assay

The wild-type (WT) and mutated (Mut) MyD88 3′UTR luciferase reporter vectors were constructed by cloning the gene sequence into a GV272-promoter vector (synthesized by Gene, Shanghai, China). The miR-149-5p mimics were also synthesized by Gene, Shanghai, China. 231/PTX cells (2 × 10^5^) were co-transfected with 0.5 μg of miR-149-5p mimics using either the GV272-MyD88-WT or GV272-MyD88-Mut construct. To monitor the transfection efficiency, the plasmid (0.05 μg) expressing Renilla luciferase was also co-transfected into each sample. After 48 h, the cells were collected, and the luciferase activity assay was measured using the dual-luciferase reporter assay system (Promega, USA).

### Tumor Xenograft Model

231/PTX cells were suspended in PBS and injected (3 × 10^6^) into the flanks of female athymic nude mice subcutaneously (6 weeks old). Ten days after injection, PTX or UA was administered. We randomized the mice into the following five groups (*n* = 6 per group): (1) control: PBS (100 μL every 3 days, ip), (2) PTX (low): PTX (10 mg/kg every 3 days, ip), (3) PTX (high): PTX (20 mg/kg every 3 days, ip), (4) UA: UA (10 mg/kg every 3 days, ip), and (5) combination: PTX (10 mg/kg every 3 days, ip) plus UA (10 mg/kg every 3 days, ip). The animals were treated for 3 weeks, and the tumor sizes and body weights were measured every 3 days. The tumor volume (V) was calculated by the formula V = L × W^2^ × 0.5 (L represents the length of tumor; W represents the width of tumor). The tumors were dissected out of the mice 3 days after the final drug administration, weighed, and photographed. The animal experiment followed the approval of the institutional animal care and use committee of Putuo District Center Hospital (Shanghai, China).

### Statistical Analysis

The data were analyzed using GraphPad Software and SPSS version 21.0 software. Student's *t*-test was used to calculate the significance, and statistically significant differences were defined as *p* < 0.05.

## Results

### UA Reverses PTX Resistance in Breast Cancer 231/PTX Cells

**Figure 1:**
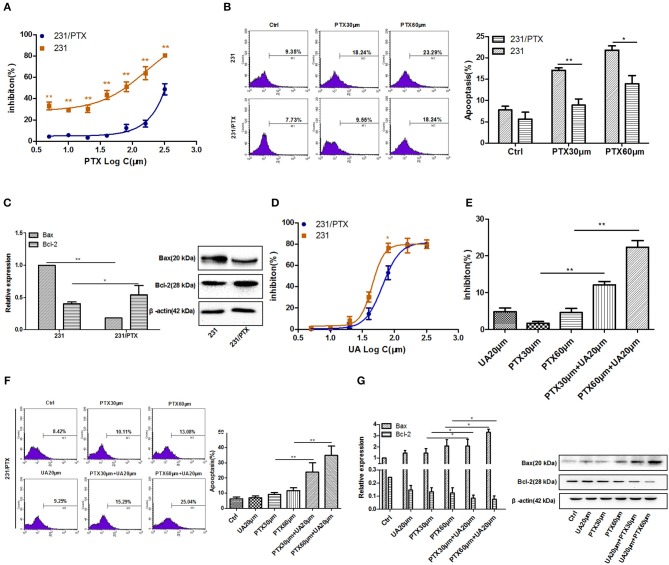
UA reverses the PTX resistance of breast cancer 231/PTX cells. **(A)** The inhibition of 231 and 231/PTX cell growth by PTX. **(B)** Apoptosis in 231 and 231/PTX cells after treatment with PTX. **(C)** Real-time PCR and Western blot analyses of the Bax and Bcl-2 mRNA and protein levels in 231 and 231/PTX cells, respectively. **(D)** The inhibition of 231 and 231/PTX cell growth by UA. **(E)** The inhibition of cell growth by PTX in 231/PTX cells after UA pretreatment. **(F)** Apoptosis in 231/PTX cells treated with PTX after UA pretreatment. **(G)** Real-time PCR and Western blot analyses of bax and bcl-2 mRNA and protein levels after UA and PTX treatment, respectively. The results (means ± SDs) were from at least three independent experiments. ^*^ *p* < 0.05 vs. the control group, ^**^p < 0.01 vs. the control group.

The PTX-resistant breast cancer cell line 231/PTX was established by Shanghai Gene Biochemistry Co., Ltd. The CCK-8 assay showed that the 231/PTX cells were significantly resistant to PTX treatment compared to the resistance of the 231 cells, as evidenced by the fact that the half-maximal inhibitory concentration (IC50) values of the 231 and 231/PTX cells, which were 74.05 and 348.96 μM, respectively (Figure 1A). Additionally, we compared the apoptosis rate between 231/PTX and 231 cells treated with PTX, and the results showed that the apoptosis rate of the 231/PTX cells was significantly decreased compared to that of the 231 cells (Figure 1B). Meanwhile, the expression of the apoptosis gene Bax was significantly decreased, while the expression of Bcl-2 was increased in 231/PTX cells compared with those in 231 cells (Figure 1C). The low-toxic concentrations (survival rate higher than 90%) of UA in these cells were determined by the CCK-8 method, and the results showed that the maximum concentrations of UA that can be used for the reversal assays can be up to 25 μM (Figure 1D).

Further, studies were performed to explore whether UA could enhance the PTX effects on 231/PTX breast cancer cells when exposed to PTX and UA individually or in combination. As shown in Figures 1E,F, at concentrations of 20 μM, UA significantly enhanced the inhibition of cell growth and augmented the apoptosis of 231/PTX cells after treatment with PTX. Furthermore, the expression level of Bax was significantly increased and the Bcl-2 level was decreased in 231/PTX cells treated with PTX plus UA compared with the levels in cells treated with PTX only (Figure 1G).These results suggest that UA could reverse PTX resistance in breast cancer cells.

### UA Reverses PTX Resistance Through the Upregulation of miR-149-5p

**Figure 2:**
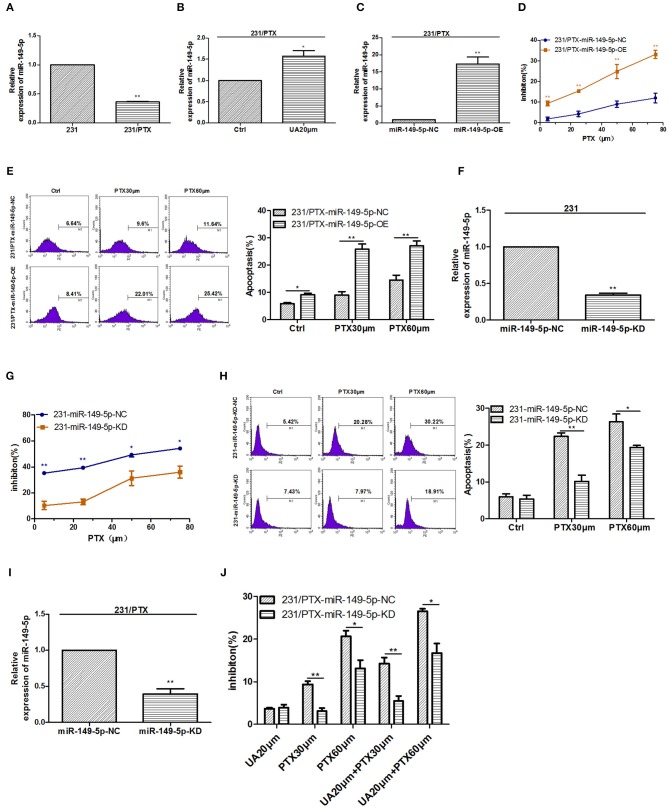
UA upregulates miR-149-5p to reverse 231/PTX cell resistance. **(A)** Real-time PCR analysis of miR-149-5p in 231 and 231/PTX cells. **(B)** Changes in the expression level of miR-149-5p in 231/PTX cells after treatment with UA. **(C)** The expression level of miR-149-5p in 231/PTX cells transfected with the miR-149-5p overexpression lentivirus. **(D)** The inhibition of 231/PTX cell growth by PTX treatment after the overexpression of miR-149-5p. **(E)** 231/PTX cell apoptosis after treatment with PTX and the overexpression of miR-149-5p. **(F)** The expression level of miR-149-5p in 231 cells transfected with the miR-149-5p inhibition vector. (G) The inhibition of 231 cell growth by PTX after the downregulation of miR-149-5p. **(H)** Apoptosis in 231 cells treated with PTX after the downregulation of miR-149-5p expression. **(I)** The expression of miR-149-5p in 231/PTX cells transfected with the miR-149-5p inhibition vector. **(J)** The inhibition of 231/PTX cell growth by PTX treatment in cells with downregulated miR-149-5p expression after UA pretreatment. The results (means ± SDs) were from at least three independent experiments. ^*^p < 0.05 vs. the control group, ^**^p < 0.01 vs. the control group.

In our previous study, we found that miR-149-5p played an important role in mediating tumor PTX resistance, so we detected the level of miR-149-5p in 231/PTX cells, and the results showed that the expression of miR-149-5p in 231/PTX cells was decreased significantly compared with that in 231 cells (Figure 2A). Furthermore, to explore whether UA reversed PTX resistance by targeting miR-149-5p in breast cancer cells, we evaluated the effects of UA on the expression of miR-149-5p, and the results showed that the level of miR-149-5p in 231/PTX cells was increased significantly compared to that in the 231/PTX control cells after treatment with UA (Figure 2B).

To confirm the efficacy of miR-149-5p on reversing PTX resistance in breast cancer cells, we used a lentivirus expressing miR-149-5p to achieve the overexpression of miR-149-5p in 231/PTX cells. qRT-PCR analysis showed that the miR-149-5p level in 231/PTX cells transfected with the miR-149-5p overexpression vector was notably higher than that in 231/PTX control cells (Figure 2C). The proliferation of 231/PTX cells overexpressing miR-149-5p in the presence of PTX was significantly inhibited (Figure 2D), while the apoptosis rate was significantly increased compared to those in the control cells (Figure 2E). Additionally, we used a lentiviral containing shRNA to specifically target and stably knockdown the expression of miR-149-5p in 231 cells. The results from real-time PCR analysis showed that the miR-149-5p expression level was significantly decreased (Figure 2F), and the inhibition of the proliferation and apoptosis rate of 231 cells was significantly decreased after treatment with PTX compared to those of the untreated cells (Figures 2G,H). These results suggest that the overexpression of miR-149-5p increased the sensitivity of breast cancer cells to PTX.NF

To further verify the efficacy of UA on the reversal of PTX resistance by targeting miR-149-5p, we transfected 231/PTX cells with a lentivirus to stably suppress miR-149-5p expression in 231/PTX cells. qRT-PCR analysis showed that the miR-149-5p level was significantly decreased in 231/PTX cells transfected with the miR-149-5p lentivirus compared with the level in the 231/PTX control cells (Figure 2I). The CCK-8 results showed that the inhibition of the 231/PTX cell proliferation in cells with miR-149-5p suppressed was decreased, compared to that of the control cells after the UA and PTX combination treatment (Figure 2J). The results indicated that UA was unable to effectively reverse PTX resistance in 231/PTX cells when the expression of miR-149-5p was suppressed. It is conceivable that UA reverses PTX resistance through the regulation of miR-149-5p expression in breast cancer cells.

### MyD88 Is a Target Gene of miR-149-5p in Breast Cancer Cells

**Figure 3:**
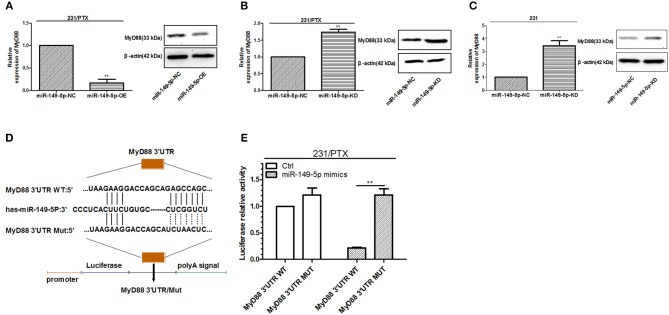
miR-149-5p targeted regulation of MyD88. **(A)** The expression level of MyD88 in 231/PTX cells after the overexpression of miR-149-5p. **(B)** The expression level of MyD88 in 231/PTX cells after the knockdown of miR-149-5p. **(C)** The expression level of MyD88 in 231 cells after the knockdown of miR-149-5p. **(D)** The miR-149-5p target sequences of MyD88. **(E)** A luciferase reporter assay in 231/PTX cells and 293 T cells. The results (means ± SDs) were from at least three independent experiments. ^**^p < 0.01 vs. the control group.

Bioinformatics analysis by TargetScan and miRanda indicated that the 3′UTR of MyD88 had binding sites for miR-149-5p. To assess whether miR-149-5p was involved in the regulation of MyD88 expression in 231 and 231/PTX cells, we detected the expression level of MyD88 by qRT-PCR and Western blot analyses. As shown in Figure 3A, the expression level of MyD88 was significantly decreased in 231/PTX cells overexpressing miR-149-5p, while the expression level of MyD88 was increased in 231/PTX cells with miR-149-5p knocked down (Figure 3B) compared to the expression level in the control cells. Additionally, there was a significant increase in the MyD88 expression level after the downregulation of miR-149-5p expression in 231 cells (Figure 3C). The results were indicative of the ability of miR-149-5p to alter MyD88 expression levels in 231/PTX and 231 cells.

To further determine whether miR-149-5p directly targets MyD88, we used a dual-luciferase reporting system with either the wild-type or mutant 3′UTR of MyD88, which contains the binding site of miR-149-5p (Figure 3D). The results showed that the luciferase activity was markedly decreased in the 231/PTX cells co-transfected with the miR-149-5p mimic and the wild-type 3′UTR of MyD88 compared with the luciferase activity in the 231/PTX cells co-transfected with the miR-149-5p mimic and the mutant 3′UTR of MyD88 (Figure 3E), which indicates that miR-149-5p can negatively regulate MyD88 expression by binding to its 3′UTR.

### Chemoresistance of 231/PTX Cells to PTX Is Reversed After the Downregulation of MyD88

**Figure 4:**
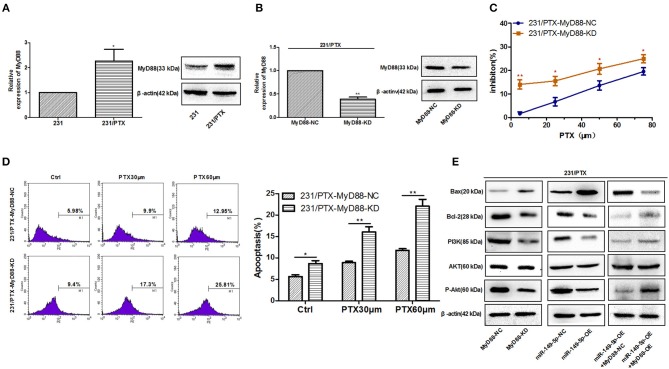
Reversed chemoresistance of 231/PTX cells to PTX after the downregulation of MyD88 expression via the PI3K/Akt pathway. **(A)** The mRNA and protein expression levels of MyD88 in 231 and 231/PTX cells. **(B)** The mRNA and protein expression of MyD88 in 231/PTX cells transfected with MyD88 RNAi lentivirus (MyD88-KD) or control lentivirus (MyD88-NC) **(C)** The inhibition of 231/PTX cell growth by PTX treatment after the downregulation of MyD88 expression. **(D)** Apoptosis in 231/PTX cells treated with PTX after the downregulation of MyD88 expression. **(E)** Western blot analysis of Bax, Bcl-2, PI3K, Akt, and P-Akt in 231/PTX MyD88-KD cells (transfected with MyD88 RNAi lentivirus), 231/PTX MyD88-NC cells (transfected with RNAi control lentivirus), 231/PTX miR-149-5p-OE cells (transfected with miR-149-5p overexpression lentivirus), 231/PTX miR-149-5p-NC cells (transfected with overexpression control lentivirus),231/PTX miR-149-5p-OE+MyD88-OE cells (transfected with miR-149-5p overexpression lentivirus plus MyD88 overexpression lentivirus),231/PTX miR-149-5p-OE+MyD88-NC cells (transfected with miR-149-5p overexpression lentivirus plus control lentivirus). The results (means ± SDs) were from at least three independent experiments. ^*^p < 0.05 vs. the control group, ^**^p < 0.01 vs. the control group.

To clarify whether MyD88 mediates the PTX resistance in 231/PTX cells, we measured the expression level of MyD88 in 231/PTX cells. The results showed that the level of MyD88 in 231/PTX cells was increased compared to that in 231 cells (Figure 4A). Furthermore, a lentiviral containing shRNA was used to specifically target and stably knockdown the expression level of MyD88 in 231/PTX cells. qRT-PCR and Western blot analyses were used to show that the expression level of MyD88 was significantly decreased in 231/PTX cells transfected with MyD88-specific shRNA compared with that in the 231/PTX control cells (Figure 4B).

We found that the proliferation of 231/PTX cells was significantly inhibited by PTX when the expression level of MyD88 remained at a lower level (Figure 4C), suggesting that the decreased MyD88 expression level could increase the sensitivity of 231/PTX cells to PTX. To confirm whether the expression level of MyD88 can change the apoptosis rate of 231/PTX cells after PTX treatment, we detected the apoptotic changes of 231/PTX cells by annexin V after treatment with PTX for 48 h, and the percentage of apoptotic cells was significantly increased for the shRNA-transfected 231/PTX cells (Figure 4D), compared with that of the control cells.

Our previous study indicated that Akt activation may be related to PTX-induced apoptosis (3). Furthermore, we investigated the roles of MyD88 and miR-149-5p in the regulation of Akt activation in 231/PTX cells. The results showed that both the knockdown of MyD88 and the overexpression of miR-149-5p greatly inhibited the activation of the Akt pathway in 231/PTX cells (Figure 4E). Additionally, the expression level of the apoptotic gene Bax was increased, while that of Bcl-2 was significantly decreased in 231/PTX cells with the suppression of MyD88 (Figure 4E). Further, our results showed that after re-overexpression of MyD88 in miR-149-5p OE 231/PTX cells, the role of miR-149-5p in regulating Akt pathway was inhibited, which demonstrated that miR-149-5p suppresses the Akt pathway by targeting MyD88 (Figure 4E).

### UA Regulates MyD88 to Reverse PTX Resistance

**Figure 5:**
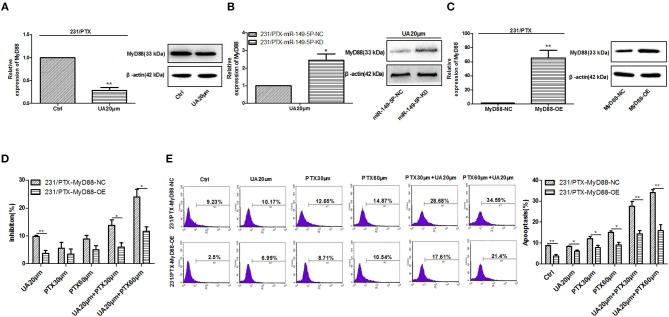
UA regulates MyD88 expression to reverse PTX resistance. **(A)** Changes in the MyD88 mRNA and protein levels in 231/PTX cells after UA treatment. **(B)** The changes in the MyD88 mRNA and protein levels were examined after the 231/PTX miR-149-5p-KD and miR-149-5p-NC cells were treated with UA. **(C)** Changes in the MyD88 mRNA and protein levels after MyD88 overexpression. **(D)** The growth inhibition of 231/PTX cells treated with UA plus PTX after MyD88 overexpression**. (E)** The apoptosis of 231/PTX cells treated with UA plus PTX. The results (means ± SDs) were from at least three independent experiments. ^*^p < 0.05 vs. the control group, ^**^p < 0.01 vs. the control group.

To explore whether UA reversed PTX resistance by targeting MyD88 in breast cancer, we evaluated the effect of UA on the expression of MyD88. The results showed that the expression of MyD88 was significantly decreased in 231/PTX cells after UA treatment (Figure 5A). Also, after down-expression of miR-149-5p, the effect of UA on decreasing the expression of MyD88 was inhibited, which indicated that UA inhibited MyD88 expression through targeting miR-149-5p (Figure 5B). It was evident that UA may regulate MyD88 to reverse PTX resistance. To verify the efficacy of UA on the reversal of PTX resistance by targeting MyD88, we used a lentivirus containing MyD88 to stably overexpress MyD88 in 231/PTX cells. qRT-PCR and Western blot analyses showed that the MyD88 expression level in the 231/PTX cells transfected with the MyD88 lentiviral was significantly higher than that in the control cells (Figure 5C). The CCK-8 results showed that the overexpression of MyD88 decreased the inhibition of 231/PTX cell proliferation after the UA and PTX combination treatment compared to that of the control cells (Figure 5D). We further explored the percentage of apoptosis by flow cytometry, and the results showed that the apoptosis rate of 231/PTX cells overexpressing MyD88 was decreased after UA and PTX combination treatment compared to that of the control cells (Figure 5E). These results indicate that UA was incapable of effectively reversing PTX resistance in 231/PTX cells with the pre-existing overexpression of MyD88. UA reverses PTX resistance through the upregulation of MyD88 expression in breast cancer cells.

### UA Reverses the PTX Resistance of Breast Cancer Cells *in vivo*

The above results demonstrated that UA reversed the PTX resistance by targeting miR-149-5p/MyD88 *in vitro*. To validate the results *in vivo*, a breast cancer xenograft model was established by subcutaneous inoculation in nude mice. We then tested the effects of PTX (10 and 20 mg/kg separately), UA (10 mg/kg), and the combination of UA (10 mg/kg) and PTX (10 mg/kg) on tumor growth inhibition.

**Figure 6:**
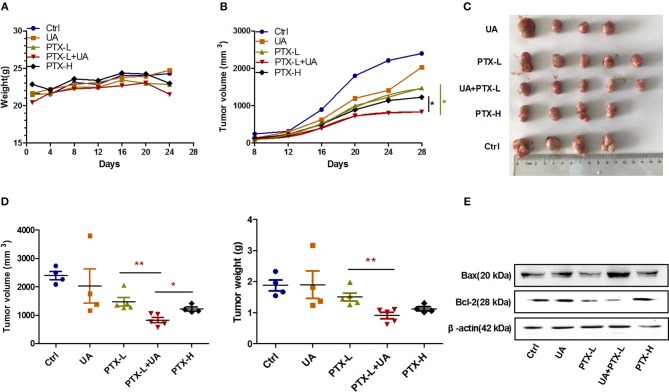
UA reverses PTX resistance *in vivo***. (A)** The growth curve of the nude mouse's body weight. **(B)** Changes in tumor volume with time after tumor cell inoculation. **(C)** Images of tumors were taken at the same scale on the 28th day after inoculation. **(D)** The tumor volume and weight on the 28th day after implantation. **(E)** Western blot analysis of the Bax and Bcl-2 protein levels extracted from nude mouse tumors. The results (means ± SDs) were from at least three independent experiments. ^*^p < 0.05 vs. the control group, ^**^p < 0.01 vs. the control group.

There were no significant differences in the body weight development of the mice in the different treatment groups (Figure 6A), while the development of tumor volume in the combination group of UA (10 mg/kg) and PTX (10 mg/kg) was slower than those in the other groups (Figure 6B). As shown in Figures 6C,D, the tumor sizes and tumor weights in the combination group of UA (10 mg/kg) and PTX (10 mg/kg) were smaller than those in the individually-treated groups. These two parameters were found to be even smaller than those in the group treated with a high dose of PTX (20 mg/kg). Furthermore, the expression level of the apoptotic gene Bax was significantly increased, while the expression level of Bcl-2 was decreased in tumor tissues treated with PTX plus UA compared with the expression levels of cells treated with PTX only (Figure 6E). Taken together, these results demonstrate that UA can enhance the *in vivo* antitumor effects of PTX.

## Discussion

PTX is a first-line chemotherapeutic agent for breast cancer treatment, but the efficacy of PTX is limited by the resistance that is inevitably acquired after long-term exposure. However, there are no biomarkers to predict the clinical responsiveness or resistance of breast cancer to PTX treatment, and the development of new molecules or drugs for breast cancer screening and treatment is urgently needed. In this study, we aimed to explore a candidate compound, ursolic acid, that can effectively reverse the PTX resistance of breast cancer cells, to elucidate its molecular mechanism.

The molecular analysis of miRNAs is helpful to develop appropriate clinical lab diagnostic methods and to understand the molecular mechanisms of chemoresistance (22, 23). It has been reported that miRNAs play a critical role in regulating gene expression during chemotherapy treatments, as miRNAs can directly target the protein-coding genes and can inhibit the genes required for signaling pathways or drug-induced apoptosis; multiple miRNAs are considered critical for the control of drug resistance (24). Previous studies have shown that miR-149 is downregulated in breast cancer tissue (25) and in many other types of cancer tissues (26–28); in addition, miR-149 plays a key role in the suppression of cancer by targeting multiple oncogenes that regulate tumor-related processes. Moreover, studies have indicated that miR-149 plays a key role in reversing chemoresistance to chemotherapeutic agents, such as cisplatin, 5-fluorouracil and gefitinib (29–31). However, no studies have reported the role of miR-149 in PTX resistance in breast cancer cells. In this study, we found that the expression level of miR-149-5p was significantly decreased in 231/PTX cells compared to that of the control cells and that a strong inverse correlation was observed between the expression levels of miR-149-5p and MyD88. In addition, our results demonstrate that the overexpression of miR-149-5p can effectively enhance PTX-induced cell apoptosis by inhibiting the expression of MyD88 and by inhibiting the PI3K/Akt signaling pathway, which suggests that this may be an effective strategy for the treatment of PTX resistance to restore the miR-149-5p expression levels in breast cancer cells.

Studies have shown that MyD88 plays an important role in mediating the development of cancer and PTX resistance (32, 33). Our previous studies have also found that the expression of MyD88 was increased in breast cancer tissues and that the sensitivity of breast cancer cells to PTX was significantly increased after the regulation of the MyD88 expression (34). In this study, we found that the upregulated expression of MyD88 in 231/PTX cells could effectively reverse the resistance of 231/PTX cells to PTX. Furthermore, to clarify the molecular mechanism of MyD88 in mediating PTX resistance, we screened for potential regulators that were previously reported in the literature; for example, it was reported that miR-149 could regulate MyD88 in macrophages (14). As expected, we concluded that MyD88 serves as a target of miR-149-5p in breast cancer 231/PTX cells, and the luciferase reporter gene experiments showed that miR-149-5p directly targeted the 3′UTR of MyD88.

PI3K/Akt is an important signaling pathway that is activated in PTX-resistant prostate and breast cancers (34, 35). Our results showed that the PI3K/Akt pathways were inhibited after the downregulation of MyD88 or the overexpression of miR-149-5p, compared to that in the control cells; in addition, our results showed that the expression of Bax was increased while the expression of Bcl-2 was decreased in 231/PTX cells compared to those in the control cells, which suggests that MyD88 mediated PTX resistance through the PI3K/Akt pathway in breast cancer cells.

To date, there are several methods to treat PTX-resistant cancers, such as treatment with microtubule-targeting agents, CDK1, autophagy inhibitors, or apoptosis modulators, or by reducing the drug efflux (36–38). However, there are currently no studies reporting the single drug reversal effects of PTX resistance in breast cancer cells. Thus, we attempted to identify and develop potential therapeutic options for PTX resistance in breast cancer with traditional Chinese monomers. UA is a pentacyclic triterpenoid derived from the berries, leaves, and fruits of medicinal plants, such as rosemary and calla (39); UA has been reported to inhibit tumorigenesis (40) and to suppress tumor angiogenesis effectively (41). In this study, we found that a safe concentration of UA significantly reversed the resistance of 231/PTX cells to PTX. To further investigate the mechanism of UA's reversal effect, we determined the expression levels of miR-149-5p and MyD88 in 231/PTX cells after treatment with UA, and our results show that UA can increase the level of miR-149-5p and can decrease the level of MyD88 in 231/PTX cells. Furthermore, the 231/PTX cell xenograft model in nude mice proved the reversal efficacy of UA *in vivo*.

In summary, we reported the reversal effect of UA on PTX resistance in human breast cancer cells and its underlying mechanisms. Our results showed that UA can effectively increase the expression level of miR-149-5p, while the overexpression of miR-149-5p can effectively reverse PTX resistance by inhibiting the expression of MyD88. Taken together, our data suggest that the UA and PTX combination therapy may be an effective strategy to overcome clinical breast cancer cell resistance to PTX.

## Data Availability

All datasets generated for this study are included in the manuscript

## Ethics Statement

This study was carried out in accordance with the recommendations of National Institute of Health guidelines for the Care and Use of Laboratory Animals, the institutional animal care and use committee of Putuo District Center Hospital, Shanghai, China. The protocol was approved by the institutional animal care and use committee of Putuo District Center Hospital, Shanghai, China.

## Author Contributions

XK, RW, and FX conceived and designed the project. FX, YF, and ZN performed the experiments and acquired the data. QL, ZZ, and ZC analyzed the data. WH and HY participated in writing the article. All authors read and approved the final version of this manuscript.

### Conflict of Interest Statement

The authors declare that the research was conducted in the absence of any commercial or financial relationships that could be construed as a potential conflict of interest. The reviewer L-JZ declared a shared affiliation, with no collaboration, with the authors FX, YF, ZN, ZZ, ZC, and RW to the handling editor at the time of review.
